# DPP-4 Inhibitors as Potential Candidates for Antihypertensive Therapy: Improving Vascular Inflammation and Assisting the Action of Traditional Antihypertensive Drugs

**DOI:** 10.3389/fimmu.2019.01050

**Published:** 2019-05-09

**Authors:** Jianqiang Zhang, Qiuyue Chen, Jixin Zhong, Chaohong Liu, Bing Zheng, Quan Gong

**Affiliations:** ^1^Department of Immunology, School of Medicine, Yangtze University, Jingzhou, China; ^2^Cardiovascular Research Institute, Case Western Reserve University, Cleveland, OH, United States; ^3^Department of Microbiology, School of Basic Medicine, Tongji Medical College, Huazhong University of Science & Technology, Wuhan, China; ^4^Clinical Molecular Immunology Center, School of Medicine, Yangtze University, Jingzhou, China

**Keywords:** DPP-4, DPP-4i, GLP-1, inflammation, cardiovascular effects, hypertension

## Abstract

Dipeptidyl peptidase-4 (DPP-4) is an important protease that is widely expressed on the surface of human cells and plays a key role in immune-regulation, inflammation, oxidative stress, cell adhesion, and apoptosis by targeting different substrates. DPP-4 inhibitors (DPP-4i) are commonly used as hypoglycemic agents. However, in addition to their hypoglycemic effect, DPP-4i have also shown potent activities in the cardiovascular system, particularly in the regulation of blood pressure (BP). Previous studies have shown that the regulatory actions of DPP-4i in controlling BP are complex and that the mechanisms involved include the functional activities of the nerves, kidneys, hormones, blood vessels, and insulin. Recent work has also shown that inflammation is closely associated with the elevation of BP, and that the inhibition of DPP-4 can reduce BP by regulating the function of the immune system, by reducing inflammatory reactions and by improving oxidative stress. In this review, we describe the potential anti-hypertensive effects of DPP-4i and discuss potential new anti-hypertensive therapies. Our analysis indicated that DPP-4i treatment has a mild anti-hypertensive effect as a monotherapy and causes a significant reduction in BP when used in combined treatments. However, the combination of DPP-4i with high-dose angiotensin converting enzyme inhibitors (ACEI) can lead to increased BP. We suggest that DPP-4i improves vascular endothelial function in hypertensive patients by suppressing inflammatory responses and by alleviating oxidative stress. In addition, DPP-4i can also regulate BP by activating the sympathetic nervous system, interfering with the renin angiotensin aldosterone system (RAAS), regulating Na/H_2_O metabolism, and attenuating insulin resistance (IR).

## Introduction

Dipeptidyl peptidase-4 (DPP-4), which is also referred to as adenosine deaminase complexing protein 2 (ADCP2), cluster of differentiation 26 (CD26), or adenosine deaminase binding protein (ADBP), is an important protease that is widely expressed on the surface of human cells. As a member of the leukocyte surface antigen family, DPP-4 plays an important role in the immune system, and can regulate inflammation, oxidative stress, cell adhesion, and apoptosis. Due to their inhibitory effects on T cell activation and function, DPP-4 inhibitors (DPP-4i) have been successfully evaluated *in vivo* as immunosuppressive therapies using animal models of rheumatoid arthritis (RA), multiple sclerosis (MS), and transplantation. Otherwise, it cleaves N-terminal two amino acids with alanine or proline in the penultimate position by way of its enzyme activity. The substrates of DPP-4 can be divided into three groups: regulatory peptide; chemokines and cytokines, and neuropeptides (1). The most well-known substrates are glucagon-like peptide 1 (GLP-1), neuropeptide Y (NPY), stromal-cell-derived factor-1 (SDF-1), substance P, and B-type natriuretic peptide (BNP) (1). In addition to catalytic functions, DPP4 also interacts with different types of ligands, including adenosine deaminase (ADA), caveolin-1, fibronectin, and C-X-C chemokine receptor type 4 (CXCR4) (1).

Due to the efficacy of GLP-1 upon blood glucose regulation, DPP-4i has gradually become a new anti-diabetic drug for the treatment of type 2 diabetes mellitus (T2DM). In addition to its activity against hyperglycemia, DPP-4i has shown beneficial cardiovascular effects including cardioprotective action, endothelial protection, and an anti-hypertensive effect. Both the EXamination of cArdiovascular outcoMes with alogliptIN vs. standard of carE in patients with type two diabetes mellitus and acute coronary syndrome (EXAMINE) study, and the Saxagliptin Assessment of Vascular Outcomes Recorded in Patients With Diabetes Mellitus-Thrombolysis in Myocardial Infarction 53 trialin (SAVOR-TIMI 53), examined the effects of DPP-4 inhibition on cardiovascular outcomes. However, these studies found no significant improvements in a range of safety endpoints for cardiovascular diseases (2, 3). Although its efficacy upon cardiovascular terminal events are not completely satisfactory, DPP-4i has shown beneficial cardiovascular benefits in many research studies, including the alleviation of vascular inflammation, the protection of endothelial cells, and the reduction of blood pressure (BP). For example, Leung et al. reported that DPP-4i could improve left ventricle systolic and diastolic function in T2DM (4). It has also been reported that alogliptin treatment results in a significant improvement of glomerular filtration rate (GFR) and left ventricular ejection fraction (LVEF) in patients with T2DM by increasing left ventricular systolic function (5). In another study, Read et al. reported that sitagliptin could remarkably improve cardiac ejection fraction (6). In addition, Jax et al. demonstrated that linagliptin treatment significantly improved microvascular function, but had no effect upon macrovascular function (7). Ida et al. provided evidence that trelagliptin treatment resulted in a visible increase of serum adiponectin level, which could regulate the function of vascular endothelial cells (8). Additional evidence has also suggested that DPP-4i can regulate BP. In the present review, describe the roles and mechanisms of DPP-4i in the improvement of hypertension, and discuss new anti-hypertensive therapies for T2DM patients or non-diabetics.

## The Role of DPP-4 Inhibitors in Hypertension

The first DPP-4 inhibitor, sitagliptin, was approved as an anti-hyperglycemic agent for T2DM in the United States of America in 2006. Since then, a range of other drugs have been developed and used clinically, including sitagliptin, vidagliptin, saxagliptin, alogliptin, and linagliptin. Compared with classical oral-hypoglycemic drugs, biguanides, thiazolidinediones, sulfonylureas, and alpha glucosidase inhibitors, patients receiving DPP-4i treatment have a lower incidence of hypoglycemic events and gain less weight. In addition to its outstanding glucose-lowering effect, DPP-4i have also shown non-metabolic functional activities, including anti-inflammatory effect and cardiovascular protection, particularly with regards to BP regulation.

Recent clinical trials and experimental studies have suggested that DPP-4i, can regulating cardiovascular function via different pathways directly, in either a direct or indirect manner. Extensive clinical studies have confirmed that DPP-4i exerts protective effects on hypertension patients. For example, sitagliptin and vildagliptin treatment could lower systolic blood pressure (SBP) independently of a reduction in blood glucose (9, 10). Some other studies showed that both SBP and diastolic blood pressure (DBP) were reduced after treatment with vildagliptin (11, 12). Furthermore, the hypotensive effect was not only limited to patients with diabetes, but also included other patients. For example, Hussain et al. found that sitagliptin significantly reduced BP in non-diabetic patients (13). Many other groups have provided evidence to support and therefore confirm this phenomenon (14). Consistent with these clinical trials, several recent studies have reported that DPP-4i can alleviate hypertensive conditions in animal models (15–19). In contrast, several studies have reported that humans or animals treated with DPP-4i do not show changes in BP when compared with placebo (4, 20, 21). Furthermore, some studies have demonstrated that a combination of DPP-4 and high-dose angiotensin converting enzyme inhibitors (ACEI) can actually increase blood pressure (22, 23). It is therefore very valuable to clarify the mechanism of BP regulation in response to DPP-4i, particularly in combined drug treatments (Table 1).

**Table 1:** The regulatory effects of DPP-4i on blood pressure in the clinical researches and animal experiments.

**Drugs**	**Subjects**	**Number**	**Duration**	**Effects**	**Date**	**References**
Saxagliptin	Humans	102	48 w	SBP ↓ and DBP ↓	2018	(12)
Sitagliptin	Humans	454	24w	SBP ↓ and DBP ↓	2017	(14)
Vildagliptin	Humans	2108	24 w	SBP ↓ and DBP ↓	2016	(11)
Sitagliptin	Humans	70	12 w	SBP ↓ and DBP ↓	2016	(13)
Sitagliptin/Vildagliptin/Saxagliptin	Humans	25	48w	No effect	2016	(4)
Sitagliptin/vildagliptin	Humans	51	12 w	SBP ↓	2016	(10)
Vildagliptin	Rats	48	4 w	DBP ↓	2016	(19)
Vildagliptin	Rats	17	1 w	SBP ↓	2015	(17)
Sitagliptin with enalapril (10 mg/kg)	Rats	12	3 w	SBP ↑ and DBP ↑	2015	(23)
Linagliptin	Rats	59	16 w	No effect	2013	(21)
Linagliptin	Mice	60	12 w	No effect	2012	(20)
Linagliptin	Rats	48	1 w	Mean BP ↓	2012	(15)
Saxagliptin	Rats	52	8 w	SBP↓ and DBP↓	2012	(18)
Sitagliptin	Rats	16	2 w	SBP↓	2012	(16)
Sitagliptin with enalapril (10/5 mg)	Humans	24	3 w	5 mg: BP↑ 10 mg: BP↓	2010	(22)

## DPP-4 Inhibitors and BP Regulation: Potential Mechanisms

Primary hypertension is a cardiovascular syndrome characterized by elevated BP, and represents the leading cause of cardiovascular disease and stroke and the primary cause of death and disease burden worldwide (24). Hypertension is caused by the interaction of genetic factors with environmental factors, although there is no unified understanding of the mechanisms involved. At present, the generally accepted mechanisms underlying hypertension are said to involve nerves, kidneys, hormones, blood vessels, and insulin resistance. In addition to these traditional mechanisms, it is widely believed that mild inflammation can lead to BP elevation and its associated cardiovascular complications. Current treatments for hypertension include angiotensin II (AngII) type 1 receptor blockers (ARB), angiotensin converting enzyme inhibitors (ACEIs), calcium channel antagonists, beta receptor blockers, and diuretics. Although the use of antihypertensive drugs has significantly improved the quality of life of patients and reduced the incidence and mortality of hypertension complications, half of all patients are still not optimistic about BP control (25). Therefore, it is particularly important to identify new candidates for antihypertensive treatment. In view of the beneficial effects of DPP-4i on blood pressure regulation, we aimed to review the effects of DPP-4i upon hypertension in association with the immune system, blood vessels, the nervous system, hormones, kidneys, and insulin resistance. We also attempt try to provide an effective strategy for anti-hypertensive therapy.

### Immunological Mechanisms of DPP-4i Upon Hypertension

Many chronic diseases are closely related to non-specific inflammatory processes, such as insulin resistance, metabolic syndrome, T2DM, and coronary heart disease, which are associated with increased infiltration and cell proliferation of immune cells and the elevated release of inflammatory mediators. Recent data suggests that innate and adaptive immune systems contribute to low-grade inflammation and play a role in the development and progression of hypertension (26).

It has been found that DPP-4/CD26 participates in non-specific inflammation by predominantly regulating the activation and chemotaxis of mononuclear/macrophages, NK cells, and T cells. Inflammatory mediators secreted from these immune cells, such as chemokines, cytokines, adhesion molecules, and reactive oxygen species (ROS), can disturb the functional activity of the vascular endothelium (27), for example, by increasing the proliferation of smooth muscle cells and participating in vascular remodeling (28). da Silva Júnior et al. suggested that DPP-4 is associated with endothelial inflammation and microvascular function, and that DPP-4 can significantly increase blood flow (29). T lymphocytes have a general role in contributing to inflammatory responses associated with hypertension (30). Early studies showed that T cells can secret and deliver AngII via the endogenous renin-angiotensin system (RAS), therefore leading to an increase in BP (31, 32). The application of DPP-4i reduces the production of cytokines controlling the proliferation of T lymphocytes (33). In view of this, we hypothesize that DPP-4 inhibitors could show potential hypotensive effects by inhibiting T cell activation and function, and reduce the secretion of inflammatory cytokines. In addition, DPP-4i also inhibits inflammation by suppressing the activation and chemotaxis of monocytes and macrophages (34–36).

It is well-known that naive T cells are stimulated by antigen presentation by antigen-presenting cells (APCs), and T cells can differentiate into different types of effector T-cells: Th1 cells, Th2 cells, Th17 cells, and Treg cells (37). Activated T cells secrete a variety of inflammatory mediators. For example, studies have shown that Th17 cells have very high expression levels of DPP-4 (38), and that interleukin 17 (IL-17) secreted by Th17 cells initiates the progression of AngII-induced hypertension (30). Saxagliptin was previously shown to inhibit the AngII-induced activation of a range of cardiac proinflammatory/profibrotic signaling intermediates, including interleukin 18 (IL-18), interleukin 17A (IL-17A), nuclear transcription factor-κB (NF-κB), and TLR4 (39). Interleukin 6 (IL-6) and tumor necrosis factor alpha (TNF-α) have been positively correlated with blood pressure (40, 41), and the inhibition of DPP-4 has been shown to down-regulate the expression of IL-6 and TNF-α (36, 42, 43). In contrast, Tinsley et al. found that interleukin 10 (IL-10), released by Treg cells, has beneficial effects in terms of reducing inflammation, ameliorating endothelial function, and lowering BP in hypertensive pregnant rats (44). Furthermore, the DPP-4 inhibitor MK0626 has been shown to increase IL-10 levels (34).

Inflammation and oxidative stress result in a series of vascular stress reactions, causing vascular endothelial dysfunction, which then leads to hypertension (45–47). The administration of DPP-4i inhibits the immune response and relieves oxidative stress (48–50). Mega et al. also found that the administration of sitagliptinin rats with T2DM reduced the levels of lipid peroxidation (51). Alam et al. suggested that sitagliptin can prevent inflammation and fibrosis of the heart and kidney by improving oxidative stress (52). Koibuchi et al. reported that the beneficial effects of linagliptin in cardiovascular injury appeared to be attributed to the reduction of oxidative stress and the downregulation of angiotensin converting enzyme (ACE) (53). Moreover, many studies were performed to explore the deeper mechanism. For example, Jo et al. showed that DPP-4i can restrain the activity of the nod-like receptor protein 3 (NLRP3) inflammasome by alleviating nicotinamide adenine dinucleotide phosphate (NADPH) oxidase 2-associated oxidative stress (43). Hu et al. further showed that the treatment of vascular endothelial cells with sitagliptin could inhibit the TNF-induced expression of vascular cell adhesion mole 1 (VCAM-1) mRNA (54). Other research showed that advanced glycation end products (AGE), and receptor of advanced glycation end products (RAGE), induces the release of ROS and stimulates DPP-4 expression from ECs by interacting with the mannose 6-phosphate/insulin-like growth factor II receptor (M6P/IGF-IIR) (55). GLP-1 inhibit AGE/RAGE-induced ROS release and inflammatory reactions via the cAMP pathway by targeting glucagon-like peptide 1 receptor (GLP-1R) (56). Consistent with this, linagliptin significantly inhibited AGE-induced ROS generation by increasing the expression of GLP-1 (55). DPP-4 inhibition via gemigliptin prevents the abnormal vascular remodeling induced by oxidative stress via the activation of nuclear factor erythroid-derived 2 (NF-E2)-related factor 2 (57). Liraglutide also exerts anti-inflammatory effects through the GLP-1R/cyclic adenosine monophosphate (cAMP) pathway via cascading cAMP-dependent protein kinase/Liver kinase B1 (PKA/LKB1), thereby increasing nitric oxide production and suppressing NF-κB (58). The activation of NF-κB can also be suppressed by DPP-4 inhibitor (59).

Investigating the regulatory activity of DPP-4 upon the inflammatory mediators and oxidative stress associated with elevated BP may provide us with good expectations for the future use of DPP-4i in the treatment of hypertension, especially in patients with inflammation. However, until now, there is still a deficiency in our understanding of the immunological mechanisms associated with DPP-4i and BP regulation. Further research is urgently required in this regard.

### Vascular Mechanisms Underlying the Effect of DPP-4i on Hypertension

Vascular smooth muscle can be affected by various physical and chemical factors, resulting in either relaxation or contraction, thus causing effect upon BP. The structure and function of large arteries and arterioles play an important role in the pathogenesis of hypertension. Endothelial cells (ECs) covering the inner surface of the vascular wall can generate, activate, and release various vasoactive substances and regulate cardiovascular function, including nitric oxide (NO), prostacyclin (PGI2), endothelin-1 (ET-1), and endothelium-derived contracting factors (EDCF). However, other, non-endothelium derived substances, can also affect vascular smooth muscle cells and endothelial cells in different pathways.

NO is a crucial physiological signaling molecule in a diverse array of organ systems, and has important anti-inflammation effects, thereby exerting effect upon cardiovascular function, including vasodilation and endothelium protection. NO is synthesized by three types of NO synthase (NOS): endothelial NOS (eNOS), neuronal NOS (nNOS), and inducible NOS (60). Recent studies have shown that DPP-4 inhibitors can increase NO levels in hypertensive models (61–63). Linagliptin, a DPP-4 inhibitor, has been reported to upregulate eNOS and restore endothelium-dependent vasodilation (64, 65). Besides the direct vasodilative effects of NO/NOS, the vasodilation caused by DPP-4i also appears to be related to GLP-1. A previous meta-analysis provided evidence that GLP-1 analogs could significantly reduce sitting SBP, suggesting that GLP-1 may be associated with BP and vascular function (66). Consistent with this, other researchers have shown that GLP-1 can induce vasodilation (67–69). The vasodilatory response of GLP-1 may occur via two different pathways: GLP-1R-dependent and independent pathways. Hattori et al. reported that the activator of GLP-1 receptor liraglutide increased eNOS phosphorylation and NO production in a protein kinase-dependent manner (58). The metabolite of active GLP-1(7-36), GLP-1(9-36), is also known to have a vasodilatory effect via the NO/cyclic guanosine monophosphate (cGMP)-dependent mechanism (68). It has also been reported that insulin which was induced by GLP-1 could affect the diastolic function of the blood vessels via the nitric oxide pathway (70). Moreover, another study reported that the vasodilatory effects of GLP-1 are independent of insulin action (71). Other vasodilatory substances, such as a-type natriuretic peptide (ANP), cGMP, and cAMP can be induced by the GLP-1R agonist exenatide (72). In addition, BNP, another substrate of DPP-4, also possesses vasodilatation activity (73). We hypothesize that DPP-4i could simultaneously enhance the concentration of activated BNP, thus promoting vasodilatation.

In contrast, another substrate of DPP-4i, neuropeptide Y1-36 (NPY1-36), has shown potent vasoconstrictive effects. NPY is a Y1-receptor agonist released from the sympathetic nerve terminals. NPY1-36 can be converted to its inactive form NPY3-36 by DPP-4 (74). Stimulation of a sympathetic nerve leads to the release of NPY which has stronger vasoconstriction properties than norepinephrine (75). NPY1-36 causes vasoconstriction via Y1 receptors, whereas NPY3-36 is a selective Y2-receptor agonist without effect on vascular tone (76, 77). DPP-4i prevents the inactivation of NPY1-36 and exerts contraction effects which are dependent upon catecholamine (78, 79). Simultaneously, NPY can inhibit the activity of vasodilator substances and enhance vasoconstrictor substances, resulting in a significant increase in vasoconstriction (80). Prieto et al. previously showed that NPY elevates Ca^2+^ influx by stimulating L-type calcium channels, leading to the secretion and potentiation of noradrenaline (81). Other studies have also shown that NPY is able to hydrolyze phosphoric inositol on cell membranes into an inositol trisphosphate (IP3) signal substance, which can promote the release of calcium ions and increase the concentration of extracellular calcium ions (82). In addition, some studies have reported that vasoconstriction is affected by the sodium potassium pump; inhibition of this pump can change the polarization of the cell membrane, thereby regulating Na^+^/Ca^2+^ exchange (83).

These studies gave us some insight into how DPP-4i causes effects upon the vascular endothelium. Further studies should now be performed to identify the precise mechanisms involved with the action of DPP-4i. We believe that the different efficacies of DPP-4i in regulating the function of vascular endothelial cell are possibly associated with the immunity of the human body, the distribution of cytokines, and the administration of drugs. The vasoconstriction of NPY is highly Ca^2+^-dependent, thus suggesting that the combination of DPP-4i and Ca^2+^ antagonists can improve the situation. And Y1 receptor antagonists could also prevent the prohypertensive effect of NPY and possibly augment the antihypertensive effects of DPP-4i.

### Neural Mechanisms of the Action of DPP-4i on Hypertension

The sympathetic nervous system (SNS) plays an important role in regulating the heart and other visceral organs. The activity of the sympathetic nerve is responsible for the physiological needs of the body when it is in a state of tension, causing constricted blood vessels, increased heart rate, dilated pupils, and reduced secretion of the digestive glands. Vasoconstriction is caused by noradrenaline activated alpha-1 adrenergic receptor released from sympathetic ganglion neurons. In contrast, Beta-1 receptors are mainly distributed in the heart, and can increase myocardial contractility, self-regulation, and conduction function. As a result of vasoconstriction and heart excitement by SNS activation, there is a consequential rise in BP.

Substance P is an important neuropeptide and acts as both a vasodilator and sympathetic activator; it can also be degraded by DPP-4. Previous research has shown that substance P is highly expressed in the heart. The expression of tachykinin precursor 1 (TAC1), the gene encoding substance P, was up-regulated when the BP is raised, indicating an involvement of substance P in high BP conditions (84). DPP-4 is a potent lyase of substance P and DPP-4i prevents the degradation of substance P from an activated state to an inactivated state. Concurrent with DPP-4 and ACEI, the intra-arterial administration of substance P stimulated the SNS (85). Further evidence was provided by another clinical study which suggested that sitagliptin could reduce BP under low-dose enalapril treatment without effects on cardiac rhythm and hormonal level, but increased hypertensive response in combination with high-dose enalapril accompanied by increasing heart rate and norepinephrine concentration. This result suggests that the SNS was activated during maximal ACEI and sitagliptin treatment (22). Consistent with the findings in humans, the same result was also found in spontaneously hypertensive rats (SHR) (86). One reasonable explanation for this is that high-dose ACEI and DPP-4i significantly reduced the degradation of substance P, due to the combined inhibition of ACE and DPP-4. Therefore, when combined with high doses of ACEI and DPP-4i, substance P may lead to sympathetic activation rather than vasodilation.

In addition, it has also been shown that BP can be elevated by GLP-1 and the GLP-1 receptor. Recent studies have demonstrated that GLP-1 and GLP-1R agonists induced a sustained elevation of BP in rodents (45, 87). This elevation in BP may have occurred as a result of the increased sympathetic activity by GLP-1 receptor activation in the central nervous system (88). Yamamoto et al. reported that centrally and peripherally administered GLP-1R agonists increased BP and heart rate by enhancing sympathetic activity in a dose-dependent manner (89). Similarly, Trahair et al. reported that intravenous GLP-1 administration attenuated the hypotensive response in healthy older individuals (90). In contrast, another study indicated that GLP-1 does not contribute to sympathetic activation (91).

In conclusion, the BP lowering effect of DPP-4i may be reduced by stimulating sympathetic activity via reducing degradation of GLP-1 and substance P when combined with ACEI, especially in high-dose ACEI. We suggest that combined treatment with agents that block the SNS could diminish the hypertensive effect of DPP-4i and that this effect may be enhanced by agents with no effect on the SNS.

### Hormonal Mechanisms Underlying the Effect of DPP-4i on Hypertension

The renin angiotensin aldosterone system (RAAS) plays a role in regulating BP. Renin is secreted from the juxtaglomerular cells in the renal afferent arteriole, converting the angiotensinogen released by the liver into angiotensin I (AngI). Angiotensin I is subsequently converted to angiotensin II (AngII) by the angiotensin-converting enzyme (ACE) found on the surface of vascular endothelial cells, predominantly those of the lungs. AngII is the major effector of RAAS, acting on AngII receptor type 1 (AT1), causing contraction of the smooth muscle of the arteriole and stimulating the adrenocortical spherical band to secrete aldosterone. AngII receptor type 2 (AT2) is generally considered to be an antagonist of the AT1 receptor, regulating the relaxation of the smooth muscle (92). Aldosterone induced by AngII promoting the reabsorption of sodium and water from the kidney, can increase water retention, and norepinephrine secretion via positive feedback from the sympathetic end of the anterior membrane, eventually leading to an increase in BP.

Recent studies have shown that DPP-4i can exert antihypertensive effects by interfering with the function of the RAAS system. For example, in a previous study, teneligliptin ameliorated hypertension and comorbid cardiac remodeling in SHR by attenuating circulating AngII (93). Treatment with liraglutide and linagliptin during AngII infusion down-regulated the AT1 receptor and up-regulated AT2 receptor expression, suggesting that DPP-4i may reduce BP via the AngII receptor-mediated pathway (94). In addition, sodium/hydrogen exchanger 1 (NHE-1), a membrane-bound enzyme, is thought to be partly mediated by AngII and involved in modulating intracellular acidity; its activation would lead to intracellular Na^+^ retention, thus enhancing Na^+^/Ca^2+^ exchange and H_2_O reabsorption (95, 96). Moreover, Kawase et al. found that teneligliptin suppressed NHE-1 expression, an effect that was enhanced by AngII, showing that DPP-4i could reduce BP by inhibiting the AngII-NHE-1 pathway (93). However, GLP-1 may also participate in regulating the function of RAAS. For example, Chaudhuri et al. showed that exenatide administration could lead to the reduction of renin, angiotensin II, and angiotensinogen in the plasma concentrations (72).

In addition, ACE inactivates substance P via the carboxy terminus. Combined treatment with high-dose ACEI and sitagliptin significantly increased BP by elevating the expression of substance P, while low-dose ACEI had no such effect (22). We suggest that when using combined treatments of DPP-4i and anti-hypertensive drugs targeting RAAS, then high-dose ACEIs should be avoided. Moreover, the important effect of the AngII receptor upon BP control should not be underestimated. We should also remember that treatment with both DPP-4i and ARB may exert synergistic anti-hypertensive effects.

### Renal Mechanisms Underlying the Action of DPP-4i on Hypertension

Various factors act upon the renal system and cause water-sodium retention, thus increasing cardiac output and peripheral vascular resistance. Water and salt metabolism can regulate blood-volume by affecting plasma crystal osmotic pressure. Increased osmotic pressure leads to elevated blood volume and higher BP. There are many active substances that can affect the excretion of water and sodium in the kidneys, including BNP, anti-diuretic hormone, and aldosterone.

DPP-4 is also expressed in the renal proximal tubular brush border, where it regulates Na+/H_2_O reabsorption (97). DPP-4i exerts diuretic and natriuretic effects by inhibiting the activity of DPP-4 (98); sitagliptin was recently shown to reduce BP by increasing sodium excretion and reducing H_2_O reabsorption (15). The hypotensive effect of DPP-4i seems to contribute to the reducing activity of NHE3, a Na+/H+ exchanger isoform responsible for reabsorption of NaHCO_3_ and NaCl (15, 99). Researches have shown that exogenous treatment with GLP-1 can induce diuretic and natriuretic effects mediated by downregulation of the activity of NHE3 (100–102). This phenomenon indicates that the diuretic and natriuretic effects of DPP-4i are mediated by preventing the degradation of GLP-1. GLP-1 could increase the urinary excretion of cAMP, suggesting that cAMP signaling pathways may participate in this process (101). Crajoinas et al. demonstrated that GLP-1 activates the cAMP/PKA signaling pathway by binding to its receptor GLP-1R and by then phosphorylating the PKA consensus site of NHE3 (101). However, Girardi et al. suggested that the effect of DPP-4i in reducing NHE3 activity results from the inhibition of a tyrosine kinase signaling pathway rather than by activation of PKA (103). The natriuretic effect of teneligliptin was partially associated with GLP-1R, and the natriuretic effect was inhibited by the GLP-1R antagonist, exendin9-39; diuresis, however, was not affected (98). Ronn et al. previously showed that these diuretic and natriuretic effects cannot be fully inhibited by the GLP-1 inhibitor exendin9-39 in SHR, which lack the expression of GLP-1 receptors (104). These findings indicated the involvement of some other unknown pathways.

BNP is a member of the natriuretic peptide family which plays an important physiological role in maintaining cardiovascular and renal homeostasis (105). It is well-known that BNP has potent diuretic and natriuretic effects. DPP-4 converts BNP1-32 into its inactive form BNP3-32 (106) and can attenuate the diuretic and natriuretic effects of BNP1-32 (107). This means that DPP-4i can enhance the diuretic and natriuretic effects of BNP1-32. Another member of the natriuretic peptide family, A-type natriuretic peptide (ANP), which also has diuresis activity, was shown to be stimulated by exenatide, a GLP-1 receptor agonist (72, 108). It has also been reported that exenatide and liraglutide induced an increased concentration of ANP through the GLP-1 receptor-dependent pathway (108). In addition, insulin induced by GLP-1 may also promote ANP secretion (72).

Generally, the diuretic and natriuretic effects of DPP-4i are associated with the reduced degradation of GLP-1 and BNP. Similarly, another natriuretic peptide, ANP, is increased via a GLP-1 pathway. Anti-hypertensive drugs, diuretics, are especially effective for elderly and obese hypertensive patients when monotherapy is not satisfactory, and can significantly reduce cardiac load. Combined treatment with diuretics and DPP-4i may amplify the effects of diuretics upon the reduction of BP and decreasing cardiac load.

### Insulin Resistant Mechanisms Underlying the Action of DPP-4i on Hypertension

Insulin resistance (IR) is considered as a pathological condition in which cells fail to respond normally to the hormone insulin (109). Recently, it is generally believed that IR is common in patients with hypertension and plays a key role in cardiovascular complications. A body of evidence has indicated that IR is closely related to the occurrence and development of hypertension (110). Insulin acts directly on the vascular tissue, including endothelial cells (111), and smooth muscle cells (112). In patients with T2DM, extended treatment with insulin can significantly improve endothelial-dependent vasodilation (113). Drugs that improve insulin sensitivity have also shown beneficial effects in the control of BP (114). Insulin improves arterial endothelial function in healthy individuals but not in metabolic syndrome patients with IR; this phenomenon demonstrates that IR may be responsible for increased cardiovascular disease risk (70).

The administration of DPP-4 promotes IR (115, 116), and insulin sensitivity has been shown to be improved when DPP-4 was down-regulated (111, 117). Furthermore, clinical research has shown that DPP-4i can improve β cell activity and increase insulin release (118), and that the long term administration of DPP-4i can improve insulin sensitivity (119, 120). For example, Smits et al. found that GLP-1-based therapies, including exenatide or sitagliptin, could significantly lower BP in the same manner as insulin therapy (121), thus suggesting that the hypotensive effects of insulin may be associated with GLP-1 (122). Chen et al. further suggested that saxagliptin could upregulate nesfatin-1 secretion and ameliorate insulin resistance (12), while other research has demonstrated that nesfatin-1 increases the secretion of GLP-1 (123). Ahren et al. suggested that GLP-1 may increase insulin release via a cAMP-dependent pathway (83). Other investigators have suggested that a NOS-related pathway may also contribute to improve insulin release. NOS inhibitors, for example, NG-monomethyl-L-arginine (L-NMMA), and asymmetrical dimethylarginine, can both improve insulin sensitivity (124, 125). With an improvement of IR, initiated by the stimulatory production of NO from the endothelium, insulin gradually exhibits its vasodilatory action (111, 117).

In summary, IR disturbs the function of the vascular endothelium and weakens the anti-hypertensive effect of insulin. DPP-4i could reduce BP by improve IR, particularly in patients with T2DM. And with the improvement of IR, the vascular inflammation can be improved, thereby reducing the cardiovascular complications.

## Conclusions

As a differentiation antigen on the surface of T cells, DPP-4/CD26 plays an important role in regulating the activation and chemotaxis of mononuclear-macrophages, NK cells, and T cells. DPP-4 inhibitors can regulate anti-inflammatory and anti-hypertensive effects by regulating the functions of these immune cells, especially T cells. We found that DPP-4i exhibits strong inhibitory effects on inflammation and oxidative stress, and a few studies provide direct evidence that DPP-4iDPP-4i reduces BP by regulating immune reactions. Moreover, the inflammatory factors regulated by DPP-4i are also closely associated with hypertension. We speculate that DPP-4i could reduce BP by regulating T cell activation, thus alleviating vascular inflammation and improving oxidative stress. However, due to the lack of research data at present, and because the mechanism underlying the action of DPP-4iDPP-4i upon BP is complex, further studies are now needed to identify the precise mechanism involved.

The substrates of DPP-4 plays an important role in regulating blood pressure. GLP-1, substance P, and BNP, commonly show a vasodilation effect, while NPY has a significant hypertensive effect. Our literature review indicated that the effect of vasoconstriction is closely associated with elevated calcium concentration. Thus, the combination of calcium antagonists may be a potent solution to resist the effect of vasoconstriction in DPP-4i treatment. In addition, the regulation of Na/H_2_O metabolism, the reduction of circulating AngII levels, and improvements in insulin resistance, may all contribute to anti-hypertensive activities. In contrast, in some situations, particularly when combined with high-dose ACEI, DPP-4i may exhibit hypertensive effects by stimulating sympathetic activity and alleviating the degradation of GLP-1 and substance P. The hypertensive effects of DPP-4i may be diminished by agents that block the SNS and enhanced by hypotensive agents with no effect on the SNS (86). Furthermore, antihypertensive drugs that do not block the SNS may exhibit an increasing BP effect of DPP-4 inhibitors by lowering basal vascular tone (86).

**Figure 1:**
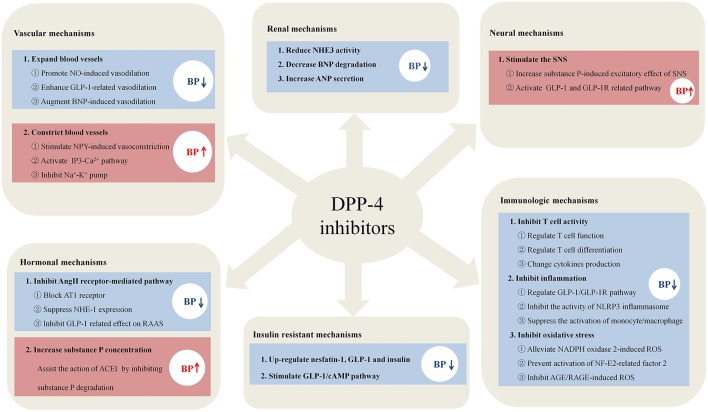
The potential mechanisms of DPP-4i in regulating blood pressure. DPP-4i has shown unique advantages in regulating blood pressure in the six mechanisms, including the aspects of immune systems, blood vessel, nervous system, hormone, kidney, and insulin resistance.

In summary, the effects of DPP-4i on BP occur in a highly context-dependent manner, and the mechanisms of DPP4-i in regulating BP are summarized in Figure 1. DPP-4i has shown good anti-hypertensive potency and combined treatment has better effect than DPP-4i alone. However, combination with high-dose ACEI could increase blood pressure. DPP-4i has shown BP modulating effects in the aspect of five traditional etiological mechanisms of hypertension, indicating that DPP-4i has great potential as a combined therapy for the treatment of hypertension; this exploits the fact that a combination of drugs causes effects on different targets. For patients with diabetes and hypertension, the advantage of DPP-4i is that it regulates blood pressure and improves insulin resistance. In addition, DPP-4i can alleviate vascular inflammation in hypertension by regulating inflammatory responses and improving vascular endothelial function, thereby reducing the incidence of cardiovascular complications. When combining DPP-4i with ARB and diuretics, the improvement of cardiac load and ventricular remodeling can be augmented. Notably, the opposite effects of DPP-4i, that promote sympathetic activation and vasoconstriction, cannot be ignored when combined with anti-hypertensive drugs. High-dose ACEI with DPP-4i may diminish the effect of anti-hypertensive drugs due to increased sympathetic activation, while low-dose ACEI has no such effect. Anti-sympathetic drugs, such as β-blockers, may represent suitable candidates in combination with DPP-4 for the treatment of hypertension with which to prevent sympathetic activity. Vasoconstriction of NPY induced by DPP-4i can similarly elevate BP via a Ca^2+^-dependent vasoconstrictive effect, thus suggesting that the combination of DPP-4i and Ca^2+^ antagonists can improve the situation.

Until now the specific etiological mechanism of hypertension has not yet been fully identified, and choosing the most appropriate and effective antihypertensive drugs is still a problem. Because it has multiple targets, DPP-4i would be a good choice with which to treat hypertension, especially in patients with chronic inflammatory diseases, such as coronary heart disease, diabetes mellitus, and hyperlipidemia. Because existing studies are still deficient, the specific mechanism of DPP-4i in regulating BP has not yet been completely clarified. Further investigations are now needed to illustrate the modulatory mechanisms and effects of DPP-4i on BP.

## Author Contributions

QG organized the article. JiaZ wrote the draft. QC drew the figure and table. BZ edited the language, figure and table. JixZ and CL revised the draft.

### Conflict of Interest Statement

The authors declare that the research was conducted in the absence of any commercial or financial relationships that could be construed as a potential conflict of interest.
